# Hedonic hunger, food addiction, and night eating syndrome triangle in adolescents and ıts relationship with body mass ındex

**DOI:** 10.1186/s40337-024-00980-7

**Published:** 2024-02-09

**Authors:** Emine Yassıbaş, Hatice Bölükbaşı, İrem Efran Turan, Ayşe Mine Demirel, Eray Gürler

**Affiliations:** Department of Nutrition and Dietetics, Faculty of Health Sciences, Gazi University, Emek, Ankara, Turkey

**Keywords:** Hedonic hunger, Food addiction, Night eating syndrome, Adolescents, Body mass index

## Abstract

**Background:**

The relationship between adolescent obesity and eating disorders is an issue that needs urgent attention. Screening for eating disorders is as important as dietary interventions to treat obesity. This study aimed to determine the relationship between hedonic hunger, food addiction, and night eating syndrome, which are considered potential risk factors for obesity, and body mass index (BMI) in adolescents.

**Methods:**

The data were collected through an online questionnaire. The “Power of Food Scale (PFS)” was used to assess hedonic hunger; the “Yale Food Addiction Scale for Children 2.0 (dYFAS-C 2.0)” was used to assess food addiction; and the “Night Eating Questionnaire (NEQ)” was used to assess night eating syndrome (NES)”. BMI was calculated using self-reported height and weight values of adolescents. The mediated structural model analysis was performed to evaluate the effect of hedonic hunger on BMI z score via food addiction and NES.

**Results:**

The study was conducted with 614 voluntary adolescents aged between 11 and 18 years. The dYFAS-C 2.0 and NEQ scores were found to be higher in adolescents with overweight and obesity, and the BMI z-score of the adolescents had significant positive correlation with their PFS scores (*p* < .001). Hedonic hunger has no direct effect on BMI independent of food addiction and NES (β = − 0.051, *p* = .468), but when the total indirect effect is evaluated with the bootstrap analysis, it was found that one-unit increase in hedonic hunger score increases BMI z-score by approximately 0.22 units (β = 0.223, SE = 0.046, 95% CI 0.131–0.313). Hedonic hunger, food addiction, and NES together explained 5.2% of the total variance in BMI z score.

**Conclusion:**

This study showed that hedonic hunger significantly predicted BMI z-score in adolescents through food addiction and NES. This emphasizes the critical importance of evaluating adolescents in terms of hedonic hunger, food addiction, and NES in the prevention, diagnosis, and treatment of obesity.

## Abstract (plain language summary)

The relationship between adolescent obesity and eating disorders is an issue that requires urgent attention. This study was conducted to determine the relationship between hedonic hunger (drive to eat for pleasure in the absence of true physical hunger), food addiction and night eating syndrome and obesity in adolescents. An online questionnaire, which assessed hedonic hunger, food addiction, and night eating syndrome, was administered to adolescents with parental consent. The study was conducted with 614 volunteer adolescents aged between 11 and 18 years. Adolescents with overweight and obesity were found to have a higher tendency to food addiction and night eating syndrome. In addition, as the hedonic hunger tendency of adolescents increased, body mass index also increased. This emphasizes the critical importance of assessing adolescents in terms of hedonic hunger, food addiction and night eating syndrome in the prevention, diagnosis, and treatment of obesity.

## Introduction

Adolescence is a critical period in the development of lifestyle and eating habits that may have various health implications in adulthood [1]. This period is characterized by significant changes in body size and composition, insulin sensitivity, eating behaviors, and psychological states, and it is considered a “high-risk period” for weight gain [2]. One of the significant health risks for adolescents is obesity, and it is estimated that 254 million adolescents worldwide will have obesity by 2030 [3]. The relationship between adolescent obesity and eating disorders is an issue that requires urgent attention [4]. Current guidelines recommend the screening of eating disorders before dietary interventions to treat obesity, and important components of clinical practice include the assessment and monitoring of eating pathologies [4, 5]. Adolescent obesity is mostly associated with Other Specified Feeding or Eating Disorder (OSFED), and binge eating is particularly emphasized in the literature [4]. Night eating syndrome (NES) is an eating disorder that is currently classified as an OSFED under the Diagnostic Statistical Manual-5 (DSM-5) [6] and it has been less researched in adolescents. Lepley, Schwager, and Khalid [7] have stated that NES is an eating disorder that requires further research, especially on the development of symptoms in adolescents and during the transition to adulthood.

NES is increasingly known due to its role in the development and continuation of obesity, is characterized by morning anorexia, evening hyperphagia, nocturnal eating, and insomnia [8]. NES is among the most common eating disorders in adolescents [9] and makes weight control more difficult [10]. Because of limited sleep activity in individuals, ghrelin levels increase; leptin levels decrease; and this situation raises not only the total amount of food intake within 24 h but also the consumption of snacks [11]. It was observed that sleep duration and quality in NES deteriorate [12], and this situation can trigger hedonic hunger [13].

Hedonic hunger is defined as taking pleasure from eating or the urge to eat, even in the absence of physiological hunger. Rather than metabolic needs, hedonic hunger leads to more energy intake for pleasure and increases the prevalence of overweight and obesity, especially in childhood and adolescence [14]. It is thought that pleasure from delicious foods may promote hedonic eating and therefore may be a major culprit for obesity. Hedonic hunger, eating behavior, food choice preference, and motivation are involved in food addiction, and food addiction is associated with increased intake, which is considered one of the main causes of obesity [15]. Foods that have high carbohydrate and fat content are more stimulating than nutritious foods. Such foods stimulate the reward mechanism in the brain, inhibit meso-cortico-limbic pathways, and reduce opioid receptors. The reduced quantity of opioid receptors increases susceptibility to hedonic eating behaviors and the preference for high-energy foods, which indirectly leads to food addiction [16, 17]. It is thought that adolescents that experience hedonic hunger turn to food intake to cope with negative emotions [18], and this situation could be associated with food addiction and NES.

There have been significant changes in the lifestyles of adolescents after the lockdowns experienced during the pandemic period [19]. Considering the current literature, it is thought that delayed bedtime, time spent at home, and increased screen exposure may pose a risk for night eating syndrome, hedonic hunger, and food addiction, and these factors may cause an increase in BMI [19, 20]. Therefore, the aim of this study was to determine the relationship between hedonic hunger, food addiction, and NES, which may consider potential risk factors for obesity, and BMI in adolescents.

## Methods

### Participants

The study was conducted between November 2021 and July 2022 in different middle and high schools in Ankara/Turkey. The school administrations were contacted beforehand, and the families were informed about the study by the administration. The adolescents whose families gave permission were included in the study. Before the online survey started, adolescents were given the option of whether they agreed to participate in the study, and if they agreed, the survey section was opened. During the administration of the questionnaire in the schools, a researcher was present in the classroom and answered the adolescents' questions in case of incomprehension.

To determine the sample size power analysis was performed using the G*Power 3.1 program and the number of individuals to be included in the study was calculated as a minimum of 296 based on a Type I error rate (α) of 0.05, a power (1-β) of 0.95, and an effect size f 0.25 [21]. The sample consisted of 614 adolescents between the ages of 11 to 18, including 270 males and 344 females. Adolescents who were receiving medical treatment due to sleep disorders and psychological disorders were excluded.

### Data collection instruments

The data were collected through an online questionnaire prepared by the researchers. The form included questions on the descriptive characteristics of the participants, such as age, sex, weight, and height. Also, to evaluate daily sleep duration, general bedtimes at night and wake-up times in the morning were questioned. The “Power of Food Scale” was used to assess hedonic hunger; the “Yale Food Addiction Scale for Children 2.0″ was used to assess food addiction; and the “Night Eating Questionnaire” was used to assess NES”. BMI was calculated using the weight and height values obtained based on the self-reports of the participants. BMI for age z-scores was evaluated using the references of the World Health Organization (AnthroPlus program).

#### Power of Food Scale (PFS)

Power of Food Scale (PFS), which is used to evaluate hedonic hunger status, was developed by Lowe et al. [22] and tested for validity and reliability in Turkish by Ulker et al. [23]. Turkish version of PFS is a 5-point Likert-type scale consisting of 13 items, each item having response options varying from “do not agree at all” to “strongly agree”. PFS consists of 3 subscales regarding food proximity, namely, food available (items 1, 2, 9, and 10), food present (items 3–6), and food tasted (items 7, 8, 11, 12, and 13). PFS total and subscale scores are obtained by summing the item scores and dividing the sum by the number of items. Higher scores indicate a higher tendency toward hedonic hunger [23]. The internal consistency reliability coefficient of the Turkish version of PFS was found 0.92. In this study, Cronbach’s alpha coefficient was found to be 0.91.

#### Yale Food Addiction Scale for Children 2.0 (dYFAS-C 2.0)

The scale assesses eating behaviors in the form of attacks in children observed for the last 12 months, considered to indicate food addiction. The final form of the scale was developed by Schiestl and Gearhardt et al. [24], and Turkish validity and reliability of the scale was conducted by Yılmaz [25]. Yale Food Addiction Scale for Children 2.0 (dYFAS-C 2.0) consists of 16 items, and all items are measured using a 5‐point Likert scale (0 = never, 1 = rarely, 2 = sometimes, 3 = very often, 4 = always). Scores on the dYFAS‐C 2.0 could range from 0 to 64, and the higher scores indicate higher levels of food addiction [24]. The internal consistency reliability coefficient of the Turkish version of YFAS-C 2.0 was found 0.90 in the original study. The Cronbach’s alpha coefficient was determined to be 0.92 in this study.

#### Night Eating Questionnaire (NEQ)

Night Eating Questionnaire (NEQ) is a scale that was developed by Alison et al. [26], which includes 14 items. The scale includes questions on the frequency of food intake after dinner, the first food of the day and morning appetite, eating in the evening and at night, food cravings, difficulty in falling asleep, the frequency of waking up at night to eat, awareness and mood during night eating, and control overnight eating behaviors. While all participants respond to the first 9 items, there is an instruction for participants who do not wake up at night to eat or have snacks to not continue to answer. Items between 10 and 12 are answered by participants who wake up at night to eat, and items 13 and 14 are answered by participants who have night-snacking habits. Item 7 questions the change in mood during the day, and those who do not experience any change in mood are given 0 points. All items except item 7 are 5-point Likert-type, each with a score range of 0–4. Items 1, 4, and 14 were inversely scored. Item 13 questions the awareness of midnight snacking behaviors, but it is excluded from the scoring. The minimum and maximum scores on the scale are 0 and 52. Two additional questions, which would constitute items 15 and 16, are recommended to be asked, but they are also not included in the scoring. In the original study, it was stated that NEQ scores equal to or greater than 25 indicate NES, and scores below 25 indicate the absence of NES [26]. The Turkish validity and reliability of the scale was conducted by Atasoy et al. [27]. The internal consistency of the Turkish version of the NEQ was found to be satisfactory (0.69) in Atasoy et al. [27] study and also in this study (0.61).

### Statistical analysis

Statistical analyses of the data were conducted using IBM SPSS Statistics 22 (IBM SPSS, Turkey). In the descriptive analyses, categorical data were used as numbers and percentages, mean, and standard deviation values were used according to the normality of the numerical data. Compliance with the normal distribution was examined by Kolmogorov–Smirnov/Shapiro–Wilk tests and histogram plot. One-way ANOVA test was used to compare PFS, dYFAS-C 2.0 and NEQ scores according to the BMI for age z-score classification groups and Tukey’s test were used to compare differences between BMI categories. Pearson correlation coefficient was used to evaluate the relationship between BMI z-score with age, sleep duration, and scale scores. The mediated structural model analysis was performed to evaluate the effect of hedonic hunger on BMI via food addiction and NES. In mediated structural model analysis, data were analyzed with PROCESS v4.0 by Andrew Hayes. Bootstrapping was used to examine the significance of indirect effects. The bootstrap coefficient and confidence intervals were determined by making 5000 bootstraps. In mediation effect analyses conducted with the bootstrap method, the 95% confidence interval (CI) values obtained as a result of the analysis should not contain the value (0) in order for the research hypothesis to be accepted. The significance level was taken as *p* < .05.

## Results

The sample of the study included 614 voluntary adolescents (M: 270; F: 344) aged between 11 and 18 years. The mean ages of the participants were 14.5 ± 1.49 years among the males and 15.3 ± 1.52 among the females. The male adolescents had a mean weight of 65.1 ± 14.67 kg, a mean height of 171.5 ± 8.6 cm, and a mean BMI of 22.0 ± 4.11 kg/m^2^, while the female adolescents had a mean weight of 56.2 ± 10.15 kg, a mean height of 162.1 ± 5.72 cm, and a mean BMI of 21.3 ± 3.49 kg/m^2^. According to the BMI for age z-score classification, 11.9% of the males and 4.7% of females had obesity (Table 1).

**Table 1:** General characteristics of the adolescents

	Male (n:270)	Female (n:344)	Total (n:614)
	$\overline{X} \pm {\text{SD}}$	$\overline{X} \pm {\text{SD}}$	$\overline{X} \pm {\text{SD}}$
Age (years)	14.5 ± 1.49	15.3 ± 1.52	14.9 ± 1.54
Body weight (kg)	65.1 ± 14.67	56.2 ± 10.15	–
Height (m)	171.5 ± 8.60	162.1 ± 5.72	–
Sleep duration (hours/day)	7.5 ± 1.29	7.4 ± 1.50	7.4 ± 1.42
BMI (kg/m^2^)	22.0 ± 4.11	21.3 ± 3.49	21.6 ± 3.79

BMI for age z-score classification	n (%)	n (%)	n (%)
Underweight (< -2SD)	6 (2.2)	12 (3.5)	18 (2.9)
Normal weight (-2SD /+1SD)	165 (61.1)	236 (68.6)	401 (65.4)
Overweight (+ 1SD/+2SD)	67 (24.8)	80 (23.3)	147 (23.9)
Obese (> + 2SD)	32 (11.9)	16 (4.7)	48 (7.8)

BMI, Body mass index

According to BMI for age z-score classification, dYFAS-C 2.0 and NEQ scores were found to be higher in adolescents with overweight than in adolescents with underweight and normal weight (*p* < .001) (Table 2). Furthermore, the BMI for age z-score of the adolescents had significant positive correlations with their dYFAS-C 2.0, NEQ, and PFS scores (*p* < .001) (Table 3).

**Table 2:** Evaluation of The Power of Food Scale, Yale Food Addiction Scale for Children 2.0 and Night Eating Questionnaire scores of adolescents according to BMI for age z-score classification

	BMI for age z-score classification (X̅ ± SD)				*p*
	Underweight (n:18)	Normal weight (n:401)	Overweight (n:147)	Obese (n:48)	
The Power of Food Scale	2.6 ± 0.85	2.9 ± 0.74	3.0 ± 0.69	3.0 ± 0.70	.143
Food present	2.9 ± 1.06	3.1 ± 0.90	3.0 ± 0.86	3.3 ± 0.98	.049
Food taste	2.9 ± 0.93	3.4 ± 0.86	3.4 ± 082	3.3 ± 0.81	.249
Food available	2.6 ± 0.98	2.9 ± 1.01	3.1 ± 0.96	3.2 ± 0.89	.071
Yale Food Addiction Scale for Children 2.0	11.3 ± 8.49^a^	19.1 ± 11.64^b^	22.7 ± 11.48^c^	21.4 ± 12.02 ^b,c,d^	< .001*
Night Eating Questionnaire	11.3 ± 4.30^a^	15.8 ± 6.94^b^	17.6 ± 6.39^c^	17.0 ± 6.11 ^b,c,d^	< .001*

ANOVA with Tukey post hoc tests. ^a,b,c,d^ For groups of different letters (*p < .001), For groups of same letters *p* > .05

**Table 3:** The correlation of BMI for age z-score with some parameters

	BMI for age z-score	
	r	*p*
Age (years)	− 0.007	.869
Sleep Duration (hours/day)	− 0.057	.162
The Power of Food Scale	0.117*	.004
Yale Food Addiction Scale for Children 2.0	0.217*	< .001
Night Eating Questionnaire	0.164*	< .001

Pearson correlation test

^*^Correlation is significant at the < .005 level

**Fig. 1:**
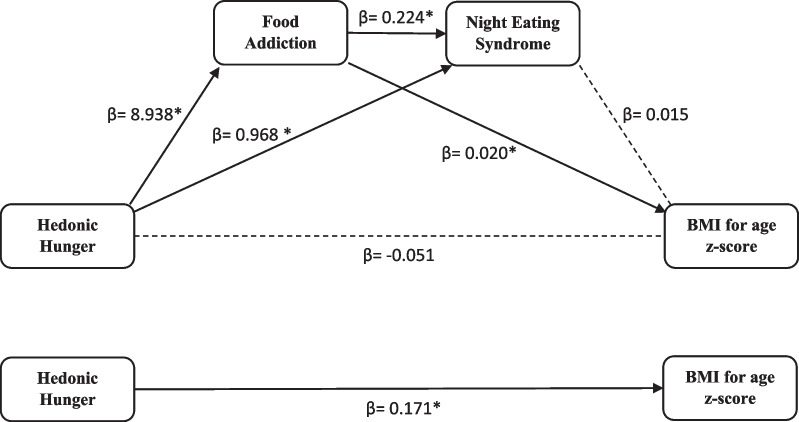
Mediated structural model analysis in which food addiction and night eating syndrome are mediating variables. *p < .05

According to the mediation analysis in the structural equation model in which food addiction and NES were the mediator variables (Table 4 and Fig. 1), hedonic hunger predicted food addiction (β = 8.938; *p* < .001) and NES (β = 0.968; *p* = .005). The coefficient of determination (R^2^) was found 37.3% for food addiction and 25.6% for NES. When the total effect of hedonic hunger on BMI z-score was evaluated, it was found that hedonic hunger significantly predicted BMI z-score (β = 0.171; *p* < .05). On the other hand hedonic hunger has no direct effect on BMI for age z-score independent of food addiction and NES (β = -0.051, *p* = .486), but when the total indirect effect is evaluated with the bootstrap analysis, it was found that a one-unit increase in hedonic hunger score increases BMI for age z-score by approximately 0.22 units (β = 0.223, SE = 0.046, 95% CI 0.131–0.313). Hedonic hunger, food addiction, and NES together explained 5.2% of the total variance in BMI for age z-score.

**Table 4:** Results of mediated structural model analysis in which food addiction and night eating syndrome are mediating variables

	Food addiction			Night eating syndrome			BMI for age z-score		
	β	SE	*p*	β	SE	*p*	β	SE	*p*
Hedonic Hunger	8.938	0.469	< .001*	0.968	0.344	**.005**	-0.051	0.074	.486
Food Addiction				0.224	0.023	< .001*	0.020	0.005	< .001*
Night Eating Syndrome							0.015	0.009	.088
Constant	− 8.478	1.536	< .001*	6.799	0.915	< .001*	− 0.047	0.203	.815
	R^2^ = 0.373			R^2^ = 0.256			R^2^ = 0.052		
	F (1, 612) = 363.516, * p* = < .001*			F (2, 611) = 105.008, * p* = < .001*			F (3, 610) = 11.172, * p* = < .001*		

Total indirect effect with Bootstrap β = 0.223, SE = 0.046, 95% CI 0.131–0.313 *p < .001

## Discussion

Obesity that begins in adolescence poses a significant public health problem because it increases the burden of non-communicable chronic diseases in adulthood. Adolescent obesity results from complex interactions between biological, developmental, behavioral, genetic, and environmental factors. The physical and psychosocial development of adolescents significantly affects their food choices and eating patterns [28]. Therefore, this study was conducted to determine the relationship between hedonic hunger, food addiction, and night eating syndrome, which are considered potential risk factors for obesity, and BMI in adolescents.

Despite the evidence supporting a relationship between hedonic hunger and the etiology of weight gain [23–25], some studies [21, 29] showed that there is no significant relationship between BMI and PFS scores. Actually, obesity-related differences in hedonic hunger have not been effectively quantified, and most of the research has not shown a linear relationship between BMI and hedonic hunger indicators. Non-linear associations with BMI have also been described for other reward-related measures like food addiction, eating disorders, and dopaminergic function [21]. In this study, it was indicated that hedonic hunger has no direct effect on BMI independent of food addiction and NES (β = -0.051, *p* = .486), but when the total indirect effect is evaluated with the bootstrap analysis, it was found that a one-unit increase in hedonic hunger score increases BMI for age z-score by approximately 0.22 units. Similarly, in the study conducted by Kaur and Jensen [30], to evaluate whether hedonic hunger predicts BMI in adolescents with overweight or obesity, it was determined that a one-unit increase in hedonic hunger led to a 0.35-unit increase in BMI z-score. These findings support the hypothesis that hedonic hunger may promote behaviors that increase the risk of increased BMI. However, while there were significant positive correlations between BMI and hedonic hunger of adolescents, it is noteworthy that there was no significant difference between hedonic hunger scores according to BMI for age z-score classification. In this instance, the lower percentage of the sample being categorized as underweight (2.9%) or obese (7.8%) may have had an impact. Also it is important to consider that hedonic hunger is not a measure of the amount or frequency of food intake, but rather assesses the disposition or motivation to eat in the absence of hunger [30]. The fact that the availability and accessibility of different foods is a crucial factor and that delicious food can often lead to thoughts and desires about food consumption, regardless of BMI, is considered important [31].

A recent meta-analysis emphasized that higher BMI could be a consequence of night eating behaviors [32]. On the other hand, some studies in the literature have reported that NES could be seen in both individuals with obesity and normal weight [33, 34]. In this study, the BMI for age z-score of the adolescents had significant positive correlations with their NEQ score (*p* < .001) (Table 3). In the evaluation of NEQ scores according to BMI for age z-score classification, it was determined that there is a significant difference between adolescents with normal weight and overweight, and there is no significant difference between adolescents with normal weight and obesity (Table 2). Although this suggests that NES may complicate eating control, it supports that it may be a triggering factor for obesity when combined with hedonic hunger.

In the literature, it is stated that as environmental stimuli and hedonic consumption of individuals increase, their food addiction scores also increase [21]. Studies have shown that there is a positive correlation between hedonic hunger and YFAS scores [21, 35]. Similarly, in this study, the BMI for age z-score of the adolescents had significant positive correlations with their dYFAS-C 2.0 score (*p* < .001) (Table 3). Furthermore, according to BMI for age z-score classification, dYFAS-C 2.0 score was found to be significantly higher in adolescents with obesity and overweight than in adolescents with underweight (*p* < .001) (Table 2). In a meta-analysis evaluating the prevalence of food addiction in children and adolescents, weight status was found to be associated marginally (*p* = .056) with the prevalence of food addiction and significantly (*p* = .002) with the severity of food addiction [36]. Therefore, for the prevention and early treatment of obesity in adolescents, it would be more useful to evaluate factors such as hedonic hunger, which may cause an increase in food intake in the development or maintenance of obesity, together with food addiction and night eating behaviors.

In conclusion, this study showed that hedonic hunger significantly predicted BMI for age z-score in adolescents through food addiction and NES. Considering that increased energy intake is one of the main causes of obesity, hedonic hunger, food addiction and NES can be considered as a factor that plays a role in the pathophysiology of obesity, and when these conditions accompany obesity, management of these conditions can be considered as an important intervention method in ensuring weight control. This underlines the need to assess adolescents in terms of hedonic hunger, food addiction and NES in the prevention, diagnosis, and treatment of obesity.

### Strengths and limitations

Both direct and indirect evaluation of the effect of hedonic hunger, which is an important factor for obesity, on BMI in adolescents and the large sample size are the strengths of this study. This study has some limitations as well as its strengths. An important limitation of this study was that the weight and height of the adolescents were recorded based on their self-reports, and BMI was calculated using self-reported height and weight values. Also, body compositions of adolescents were not examined. Other limitations include the fact that the sleep quality and the daily energy intake of adolescents were not examined. In future studies, taking food consumption records, evaluating sleep quality, and performing body composition analyses will be useful in explaining the relationship between hedonic hunger, food addiction, NES, and obesity.

## Data Availability

The datasets generated during and/or analyzed during the current study are available from the corresponding author upon reasonable request.
